# Positive Massage: An Intervention for Couples’ Wellbeing in a Touch-Deprived Era

**DOI:** 10.3390/ejihpe11020033

**Published:** 2021-05-23

**Authors:** Sayuri M. Naruse, Mark Moss

**Affiliations:** Department of Psychology, Northumbria University, Newcastle upon Tyne NE1 8ST, UK; mark.moss@northumbria.ac.uk

**Keywords:** couples wellbeing, positive massage, touch-deprivation, selves-care, stress, prevention, educational programme, COVID-19

## Abstract

COVID-19 has brought not only fear and anxiety, but also legitimate restrictions of communication and consequential touch-deprivation in our daily lives. Couples’ relational wellbeing continues to be impacted by these COVID-19 related stressors. Protecting both personal and relational wellbeing is therefore particularly important at this time. Using a preventative intervention approach, the current paper argues the theoretical benefit of the Positive Massage programme and reports a qualitative analysis of stressed but healthy couples’ experience of engaging in the programme. Thirty-four participants completed 3 weekly classes and home-based practice of massage exchange. Data from an open text online questionnaire completed every week of the programme and again 3 weeks afterwards were analysed using thematic analysis. The identified themes included “holistic stress relief”, “relationship-promotion”, and “selves-care skill”. Couples perceived Positive Massage as an effective mutual support skill to relax and help one another by de-stressing, both emotionally and physically through verbal and nonverbal communication, creating gratitude, deeper connection and self-efficacy via quality time together and pleasurable touch. Theoretically and experientially, Positive Massage can be an effective preventative selves-care skill. Promoting the concept of selves-care and its practical application through Positive Massage would be beneficial for couples’ personal and relational wellbeing in a touch-deprived era.

## 1. Introduction

Touch-deprivation is an acutely understudied but potentially critical social/behavioural/psychological/physiological health issue [1,2,3], due to it being linked with developmental delays [4,5], depression [6], aggression [7], and self-harm behaviours [8]. The modern world has become susceptible to touch-deprivation due to the fast growth of virtual communications (Field et al. reported a naturalistic observational study at airport gate waiting areas that indicated the existence of a certain form of touch-deprivation as 68% of their waiting time was spent on mobile/cell phones [9].) and the social “taboo against touch” e.g., fear of breaking the laws regarding physical contact in schools [10]. To make matters worse, the recent COVID-19 pandemic has instilled a collective fear towards touch via the restriction of communication as a consequence of social distancing and isolation [1]. These restrictions and deprivations can cause psychological harm because the human need to connect is disrupted [11]. Therefore, we would like to argue we are currently living in a “touch-deprived era”. By touch-deprived era, we refer here to the ongoing risk of deficient interactive interpersonal touch being increased due to societal state or psychological barriers such as restriction, social distancing, and fear. Field and colleagues pointed out the surprising paucity of research into touch-deprivation in the COVID-19 literature [9]. According to their survey, 60% of a sample of 260 individuals experienced low to high levels of touch deprivation during the pandemic. This is surprising because only 23% were living alone, indicating that people who live with others still felt touch-deprivation to some degree [9]. Their correlational analysis showed the link between touch-deprivation and stress, anxiety, depression, and sleep-disturbances [9].

Fortunately, couples are not included in the legitimate restrictions unless one partner tests positive for COVID-19, but an intended (e.g., deliberate distance to reduce the risk of virus transmission) and unintended consequence might be a reduction of caring touch between loved ones. Moreover, relationship science has informed us that COVID-19-related external stress has impacted and will likely continue to impact couples’ relationships negatively or positively [12,13]. While positive elements can be protected and even increased with effort in dyadic relationships, harmful dyadic processes such as withdrawal and less responsive support may be increased by the pandemic [12,13]. Based on the work by Karney and Bradbury [14], Pietromonaco and Overall [12] developed a framework explaining how the COVID-19 pandemic may shape relationships. External stressors such as economic strain, confinement, and isolation can create a context that decreases couples’ ability to give responsive support, affection, and warmth to each other because of the depletion of personal resources and self-regulation. The most negative consequences reported include an increase of intimate partner violence [15,16], anecdotal evidence of increased separation and divorce, and the prediction of not only a UK [17] but also a global increase of these consequences in the future [18].

Interestingly, accumulating social and neuroscience literature has revealed that warm touch has a positive correlation with higher levels of wellbeing, and is associated with enhanced affection in partners [19,20]. Similarly, pleasant touch seems to have a role in prosocial connection [21] such as the alignment in the physiological response of two people [22] and improvements in relationships, with examples of this available in [19,23]. Moreover, interpersonal touch can benefit couples by communicating a willingness to support, providing comfort, a reduction of stress, and demonstrating affection and care [24]. This is possibly because warm positive touch impacts the physical body directly in such ways as to lower systolic blood pressure, cortisol, and alpha amylase, and increase oxytocin [25]. Touch is one of the most instinctive and natural yet powerful ways to connect and communicate [2,26,27].

Massage is one of the most effective forms of quality touch, an intentional tactile behaviour that delivers support and care for both the giver and receiver [28]. Historically, massage has been used for healing, comforting, and caring since the time of Hippocrates (460–377 B.C.E) and is continued today in many cultures of the world [29]. Although massage has been scientifically investigated for its effectiveness in physical and psychological healing, and the benefits of massage are recognised in terms of a multidimensional professional intervention, lay people can also provide effective massage.

A lay massage i.e., a massage that is delivered by a partner, care-giver, or significant other who is not professionally trained but has received some brief instruction, has been shown to deliver various positive effects on health and wellbeing psychologically, relationally, and physically. Physical effects of lay massages include decreased pain [30] and symptoms in cancer patients [31,32], leg and back pain in pregnancy [33], and maternal pain during and post labour [34]. Reported psychological effects include the reduction of stress, anxiety, and fatigue in cancer patients [30], improved family stress and functioning [32], reduced anxiety [33,35,36] and depression in pregnant women [33,37], and improved moods in perinatal women [36]. Relational effects of massage include enhanced closeness and connection [31], marital adjustment [35], and improved relationship [33].

It is worthy to note that massage in all these investigations was one-way (i.e., from a giver to a receiver) only. A small number of distinctive studies have demonstrated that mutual massage (i.e., exchange of massage between individuals) can positively affect both the giver and receiver in terms of stress and wellbeing [28,38].

However, as far as the current authors are aware, almost no attention has been paid to quality touch or mutual massage as an intervention against the psychological effects of touch deprivation. Therefore, we propose an innovative relationship-focused intervention, Positive Massage [28], to mitigate the impact by promoting couples’ personal and relational wellbeing. The current paper aims to explore the possible effectiveness of Positive Massage for couples’ wellbeing during touch deprived periods such as the Covid-19 pandemic period and beyond. The current article focuses on couples’ personal and relational wellbeing from a preventative perspective, protecting against some of the negative psychological impacts of the COVID-19 pandemic. Maintaining or increasing the quality of relationships through mutual support and the provision of comfort and security are crucial for personal physical and emotional wellbeing [39,40], something which is especially important during this difficult time. We first describe the theoretical construct of Positive Massage and then report the experiences of couples who undertook the Positive Massage Program. We then discuss the theoretical fit of the couples experiences of Positive Massage within the framework of Covid-9 mitigation strategies [12].

Positive Massage (PM) is an innovative form of mutual massage aimed at the promotion of wellbeing in close relationships [28,38]. It is innovative, because it is not ‘receiving’ or ‘giving’ only, but involves exchanging massage in order to benefit both parties, and is based in the home setting rather than in specialised public facilities. PM was specifically designed for promoting wellbeing in the healthy general population rather than remedial use. However, the massage giver’s attention may be given to the alleviation of mild discomfort or pain when present.

The theoretical construct of PM has been addressed previously, and provides evidence that PM can be an effective positive intervention for couples’ personal and relational wellbeing [29], through the mediation of personal resources (i.e., relational, psychological, physical, and physiological). The model suggests that couples would benefit when personal resources are enhanced by PM so as to surpass the challenges (e.g., stress, time, energy, effort, and commitment). PM is a self-regulated modality that has the specific characteristics of accessibility, adaptability, and brevity. Person based features such as skills, confidence, motivation in the ‘giver’, efficacy beliefs, and relationship level would also moderate the effects. Couples who are motivated, possess skills, confidence, and a more trusting relationship are predicted to gain more benefit from PM.

One of findings of the first studies investigating PM effects indicated that both ‘receiving’ and ‘giving’ massages may elicit positive effects on emotional stress and mental clarity [28]. This interesting finding inspired the researcher to create the neologism ‘selves-care’, which refers to a health activity that cares for both a loved one and the self simultaneously [28]. The positive results of a subsequent study of PM on stressed couples’ mental wellbeing and perceived stress and coping fortified the notion of PM as a possibly effective activity for *selves-care* [38]. Moreover, based on a proposed theoretical link, PM was suggested to be a durable psychosomatic dyadic positive activity intervention in the context of positive psychology [29]. PM shares much common ground with positive psychology in the positive approach to increasing wellbeing rather than focusing on disease. Positive psychology has advanced the scientific understanding of human wellbeing, especially how and what makes people happy, promoting numerous interventions with a general target of the individual [41,42]. Among them, PM possesses a unique position not only as a dyadic approach but also as a bridge between positive psychology and massage [29].

We believe that it is very timely and useful to report the experiences of PM using previously unpublished qualitative data from the earlier study [38]. The aim of the current qualitative report is therefore to explore and inform how stressed couples experienced exchanging massages. To the authors’ knowledge, the current article is the first to report the experience of such lay mutual massages.

## 2. Materials and Methods

### 2.1. Intervention

**Figure 1:**
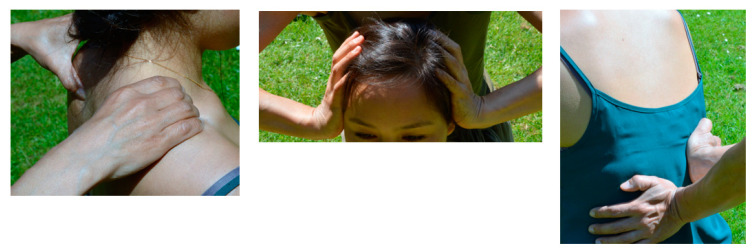
An example of a PM sequence. Left: Kneading shoulders, Middle: Circular pressing on head, Right: Palm pressing on back.

The intervention was the PM programme, made up of 1 h weekly classes coupled with the practice of PM three times per week at home. PM was contrived originally to support older people’s health and wellbeing in the local community. Therefore, the massage sequence is short, simple, user-friendly, and accessible. It requires no oil application, removal of clothing or use of a massage table [28]. The style of massage is a fusion of Western and Eastern techniques such as stroking, kneading, pressing, squeezing, tapping, and chopping. Participants are guided through a 15 min PM sequence covering massage on the back, arms, neck, shoulders, head, and face. The instructions in the programme consist of indication and contraindication, awareness practices to increase attentiveness and sensitivity, and massage skills such as T-ten (A new term that was coined by the first author. It refers to the relevant points to apply pressure, including the trigger point, acupressure point, tender point, and tight point. The term *T-ten* was created for massage givers to focus on the receiver’s body and to sense the right point to apply pressure.) as well as guidance on the use of pressure. The guided pressure ranged from gentle to moderate with some deep pressure, dependent on the technique, the target body part and the receiver’s preference. The instructions included communicating within couples to assure the partner’s preference e.g., if the partner is happy to receive a face massage due to wearing make-up. Please see the example of a PM sequence below (Figure 1) and refer to the previous articles [28,29,38] and the PM website (www.positivemassage.org, accessed on 20 May 2021) [43] for more details of the PM programme.

### 2.2. Samples

Healthy (i.e., not under medical treatment) but stressed (i.e., self-reported general stress) couples aged 18 years or more were recruited via posters, flyers, email, and social media. Exclusion criteria were: (a) known cardiovascular pathology such as an aneurysm, unless the participant had written doctor’s approval to take part in the study; (b) major surgery within last three months; (c) recent injuries such as bone fractures in the upper body; (d) being in the first trimester of pregnancy; and (e) already receiving massage regularly. Forty-eight participants i.e., 24 couples, provided informed consent. Out of these, 42 participants started the study and 38 completed it. Only 34 participants’ data were analysed due to insufficient data records. Participant couples were randomly allocated to two groups: Group A received the immediate PM intervention; and group B were assigned delayed PM intervention. Qualitative data was collapsed across the groups for the aim of the current report. Participants’ mean age was 36.8 (SD = 10.3), and out of 17 couples, 14 couples were heterosexual. Table 1 illustrates the details of participants’ characteristics.

### 2.3. Procedure

The study was approved by the Faculty of Health and Life Sciences Research Ethics Committee at the University of Northumbria. Following informed consent and agreement, couples in both groups A and B were invited to the three-week PM massage course at Northumbria University in weeks 1–3 and weeks 4–6 respectively. The PM programme was delivered by the first author and supported by an assistant. Following instruction, participants were asked to practice both giving and receiving a 15 min massage three times weekly for three weeks. Participants were permitted to change the massage duration and the target parts of the body according to the receiver’s preference.

The qualitative data was collected online via Qualtrics software (www.qualtrics.com, accessed on 20 May 2021) [44]. Participants completed the survey in their homes each week for the three weeks of the massage course, and three weeks after the completion of the massage course for group A only. The survey questions consisted of open questions such as “Do you have any comments about your partner’s massage?”; “Is there anything else you would like to let your partner know about their massage?”; “What are the benefits of PM?”; “What are the disadvantages of PM?”

### 2.4. Data Analysis

**Figure 2:**
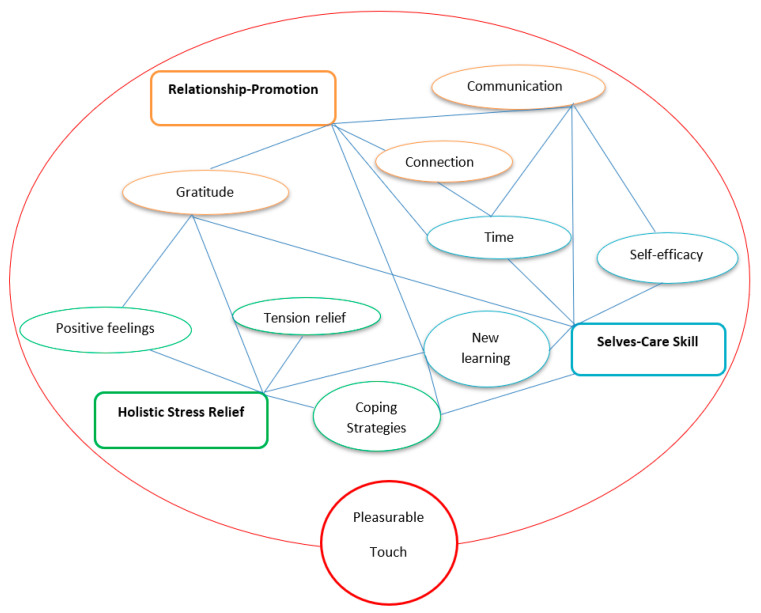
Final Thematic Map: Couples’ experiences of Positive Massage.

The collected data were analysed using thematic analysis following the six steps of Braun and Clarke’s method [45]. We took an inductive approach to identify couples’ experience of the intervention, adopting the epistemological view of critical realism [46]. This is because the authors believe that knowledge may be changed in the light of new data and fresh understanding, and as such the correct understanding of the participants responses is critical. After a number of readings of the responses to the questionnaires (phase 1), *complete coding* was performed to generate the initial codes (phase 2). Data was then collated relevant to each code (phase 3) to create the initial thematic map (phase 4). The themes were further reviewed as the thematic map was developed (Figure 2) where each was defined and named (phase 5). The results of identified themes are reported as follows (phase 6).

## 3. Results

The thematic analysis identified three superordinate themes in the couples’ experiences of PM throughout the programme:

(a) Holistic stress relief: Strategies for emotional, mental, and physical tension relief by relaxing one another through caring touch;
(b) Relationship-promotion: Improved relationship via verbal and nonverbal communication using pleasurable touch that promotes positive feelings such as gratitude, love, care, and deeper connection; and
(c) Selves-care skill: Effective mutual support techniques gained by learning together through committed quality time, which can elicit a sense of self-efficacy.

The themes identified here are distinctive and yet related to each other. Although three themes were identified, it was almost impossible to separate statements that were expressed by some participants e.g.,

*“That helps not only to relax, to aliviate (alleviate) pain or to cope with stress, it also helps to improve communication with your partner, to help him or her to relax and share and enjoy a lovely time together.” (P8 D4 F 40′s)*

(In the parentheses, P = participant code, D = dyad code, M = male, F = female and age range are indicated. Quotation marks (word, a sentence or paragraph) indicate direct reference to participants’ responses.)

However, we identified *pleasurable touch* as the core value of mutual massage because it underlies each identified theme and encompasses all the perceived effects of PM. Pleasurable touch here refers to positive enjoyable feelings created through tactile stimulations by the partner. The expression of pleasurable touch such as *“feel good”, “enjoy”, “happy”, “relaxing”*, and *“loving touch”* were interwoven in all the themes and hence were indivisible. The majority of couples experienced positive feelings of pleasurable touch during PM not only when receiving but also when giving massages:

*“I feel more relax, I feel my husband and I make some time just for us and make him feel good with a massage giving from me, makes me feel happy.” (P8 D4 F 40′s)*

*“Feel more relaxed and gets rid of aches and pains. I feel very close to my partner… Also it’s nice to find t-tens on partner and help them go away.” (P18 D10 F 30′s)*

The findings are presented below by each theme. (Note that ‘couples’ does not mean that the data are provided by the couples as a unit; all the data was collected at an individual level)

(a) Holistic stress relief

The couples described experiencing *holistic stress relief* during the PM programme. *Holistic stress relief* here refers to reduced stress manifest as increased relaxation and comfort emotionally, mentally and physically. The expressions of positive feelings included *“comfort”, “relax”, “feel good”, “lift the mood”* and *“pleasure”*, and direct references to mental stress e.g., *“(The partner’s massage helped) not to worry”, “forget stressfulness”* and *“calmness”*. Physical de-stress was reflected in reduced physical discomfort and associated benefits e.g., *“gets rid of aches and pains”, “tension relief”* and *“help to sleep”.* In sum, PM was perceived to contribute to helpful *“coping strategies”* in a stressful life.

*“The massage helped me to relax and helped with the chronic tension in my neck, shoulders and upper back.” (P2 D1 M 40′s)*

*“I often feel more relaxed after the massage. It gives me time to stop and not think about anything, not worry about doing things.” (P29 D16 F 20′s)*

*“Relaxation - mental and physical. Taking a break from stressful situations.” (P27 D14 M 30′s)*

Participants experienced relaxation to the extent of both feeling drowsiness and mental clarity, which may sound contradictory but can also be viewed as harmonizing:

*“I love the fact that the massage gives me clarity but also make me drowsy.” (P5 D3 F 20′s)*

*“I gain clarity and great night sleep. It works on headaches and shoulder aches.” (P5 D3 F 20′s)*

Thus, stress relief was experienced as holistic, not only physically and mentally but also being beneficial for relational wellbeing.

(b) Relationship-promotion

A number of couples commonly experienced PM as a modality of *relationship-promotion*, which refers improving interpersonal relationships through pleasurable touch. They reported the experience of such relational improvement, thus:

*“Breathing together feels like an important moment of connection.” (P12 D7 F 20′s)*

*“Feeling in touch with each other’s mood. relaxing together.” (P26 D14 F 30′s)*

*“It is good to connect with her like this, and it seems to lift both our moods.” (P21 D11 M 50’s)*

The connection seems to become deeper during PM, and couples appreciated the closeness. Such enhanced closeness felt within couples may be linked with spending quality time together:

*“I believe we are becoming more connected to each other spiritually and emotionally through positive massage.” (P31 D17 F 40′s)*

*“I feel very close to my partner. It really makes us much closer. Its (It’s) good to spend time just the 2 of us with no interruptions. Feel very connected.” (P18 D10 F 30′s)*

*“Feel closer and improves our communication.” (P11 D6 M 20′s)*

However, during the process of learning PM, some experienced frustration towards a lack of communication:

*“if he could ask me how’s the pressure etc during the massage more often, it would be better.” (P14 D8 F 50′s)*

*“It took a while for (the partner’s name) to listen to where I said it hurt as he wanted to follow the instructions to a tee (T-ten).” (P16 D9 F 20′s)*

On the other hand however, some couples including the latter participant experienced improvements in communication whilst others reported a new level of communication during PM:

*“I have found it easier to tell him if something hurts without worrying I will hurt his feelings which is good for us.” (P16 D9 F 20′s)*

*“Communication on a new level.” (P12 D7 F 20′s)*

*“Creates opportunity to spend time together for a specific purpose which helps communication.” (P20 D11 F 40′s)*

Many expressed gratitude and appreciation. Couples valued their partners’ willingness to learn and practice the new skills of massage. Gratitude was expressed not only towards the partner and the touch, but also regarding the experience itself and *“quality time together”*:

*“I’m very proud of him for taking part and engaging in the homework.” (P31 D17 F 40′s)*

*“Loving in his touch.” (P16 D9 F 20′s)*

*“a chance to take time for each other.” (P20 D11 F 40′s)*

*“Feeling very appreciative of the moment.” (P12 D7 F 20′s)*

The act of exchanging massage was also recognised as a way to *“show love and care for each other”* and to promote *“trust and love”*:

*“(The partner) Really cares about making me feel relaxed and loved while massaging me” (P17 D9 M 20′s)*

*“It feels like we are a team and both care for each other” (P30 D16 M 30′s)*

Additionally, this demonstration of love through receiving and giving PM may have a balancing effect for couples’ relationship dynamics:

*“Also it’s nice to receive something, as I often feel secondary in the relationship.” (P29 D16 F 20′s)*

*“you appreciate your partner energy towards the massage and want to give it back.” (P34 D18 F 20′s)*

Although there were no distinct differences in the experiences between males and females in the other themes, in this theme, predominantly females expressed more regarding relationships. It is also noteworthy that some dyads (D9, D11 and D16) expressed their positive experiences in a similar way.

(c) Selves-care skill

Compared to one-way massage, probably one of the most distinctive themes identified was that of the *selves-care* [28] skill, the mutual support techniques that can relax the partner and help each other through quality time together. Stressed couples appreciated the *“new”* learning experience together and gaining a *“lifelong skill”* to *“support each other”* which was expressed as *“great massage techniques for a busy life”*. Learning new skills can of course be challenging and frustrating at first e.g.,

*“Difficulty finding T-tens” (P6 D3 F 50′s)*

*“I wish that once we find a pain point he didn’t keep losing it.” (P20 D11 F 40′s)*

However, the learning processes were perceived positively by the others as they enjoyed the improvements:

*“Gets better every time we practice. Starting to be better about identifying t-ten spots.” (P7 D4 F 40′s)*

*“There has been gret(great) improvement in skill.” (P5 D3 F 20′s)*

*“Good listening, good improvement at finding pressure Points.” (P13 D7 M 30′s)*

The learning was perceived as not being limited to massage skills but about the importance of sensitivity, understanding each other and the awareness of the need for communications:

*“She learns and improves fast because she is very sensitive” (P30 D16 M 30′s)*

*“Can sense that my partner finds it harder to give a massage when she is tired which can effect how relaxed you feel.” (P27 D14 M 30′s)*

*“It’s good, but sometimes I wish he was more sensitive to what I like, however perhaps I should be better at telling him what I like too.” (P29 D16 F 20′s)*

Generally, participants perceived the PM programme as an *“interesting way to learn”* something that *“everyone can do”,* e.g.,

*“Easy to follow step by step guidance Great skill to learn” (P7 D4 F 40′s)*

*“It is an enjoyable and accessible form of massage that was easy to learn.” (P2 D1 M 40′s)*

*“It’s hugely rewarding and fun.” (P13 D7 M 30′s)*

*“It’s a really good skill to learn.” (P18 D10 F 30′s)*

Further, the new skills seemed to provide pleasure and confidence to couples when giving massages.

*“It makes me joyful to see I can improve someones (someone’s) day by giving them positive massage. It also helps me build my confidence.” (P5 D3 F 20′s)*

*“(regarding partner’s massage) It’s pretty good, I’m happy to feel that he’s enjoying it (giving massage) as well.” (P29 D16 F 20′s)*

These improvements in skills imply increased self-efficacy in the *selves-care* skills. The descriptions of the learning and practicing experiences included *“interesting”, “pleasure”,* and improved *“confidence”*, all of which are indicators of self-efficacy [47]. Contrarily, negative perceptions also existed. Interestingly, both dyads (D1 and D14) expressed the time pressure in a very similar way:

*“Sometimes it was hard to find a time, or doing massage meant that evening work didn’t get done.” (P1 D1 F 40′s)*

*“It takes time and both my partner and have taken on more work than we have time for. So, while we enjoy the massage, it means tasks have not been done and they still need completing.” (P2 D1 M 40′s)*

*“It’s difficult to find the time, which puts pressure to do the massage. I find it stressful to do the massage with a time pressure.” (P26 D14 F 30′s)*

*“Can be difficult to find time. One person may want to give or receive a massage whilst the other may not be in the mood. Partner got frustrated if I couldn’t do a certain technique correctly or comfortable.” (P27 D14 M 30′s)*

Acquiring new skills and increasing self-efficacy often requires time. The learning and practicing of PM were mostly perceived as *“quality time together”* and might have reduced any perception of effort e.g.,

*“Feeling a connection between us, when at times we lose sight of this in our busy lives Having the quiet time together without distraction as it doesn’t often happen” (P6 D3 F 50′s)*

*“Creating time for each other. Relief of stress and it always seems to improve physical feelings.” (P21 D11 M 50’s)*

*“It’s a really good skill to learn. It also really helps with feeling connected to your partner by spending quality time together.” (P18 D10 F 30′s)*

*“That it is good to take time to take care of yourself and other people.” (P30 D16 M 30′s)*

## 4. Discussion

The results of this study demonstrate that the couples perceived learning new skills as a largely positive experience (e.g., interesting and useful) but that practicing PM at home can be challenging due to lack of time or a deficiency of skill at an early stage. However, most couples appreciated the quality time together and the benefits of PM that are elicited by *pleasurable touch*. *Pleasurable touch* was the most predominant perception in experiencing PM, as it encompassed all the themes and subthemes. This supports previous research that identified significantly greater pleasure experienced from a 10 min foot massage delivered by hand compared to one delivered by a machine [48]. The mechanism of such touch based pleasure has been explained by the somatosensory pleasure circuit from the skin to the brain that includes specialized c-fibre afferents that code for the pleasurable properties of touch [49]. In addition, warm touch among married couples has been shown to decrease cortisol and increase oxytocin [25]. Although massage is clearly a form of touch, the difference between touch and massage is huge. Intensity and length as well as intention e.g., to support, to care, or to relax, are stronger, longer, and clearer in massage [50]. Therefore, the effect of massage is speculated to be stronger than daily instant touch, and pleasurable touch possibly impacts deeper than casual unintentional touch.

This pleasurable touch in PM is quite likely asexual. Although sexual pleasure is an important aspect of couples’ relationship satisfaction, PM is deliberately aimed to not induce arousal or sexual intimacy (One of the reasons is that the creator intended to change the distorted public view of massage in the UK solely as a sexual technique or pornography [51] in the western world. Although massage can be sexual [51] it can also be completely asexual, as most massage literature aims to heal or for promoting wellbeing, e.g. [31,32]. PM was originally developed as an asexual health intervention for close relationships such as family and friends more than a decade ago when massage therapists were misunderstood commonly.). However, COVID-19 has impacted indirectly on sexual functioning and has caused sexual health implications among couples [52,53]. For such couples who seek specialist help, the sensate focus method seems to be an effective intervention [54]. The sensate focus method is a structured touching developed by Masters and Johnson using a focus on mutual tactile sensations but with restrictions not to touch sexual organs at an early stage [55]. Similarly, although the aim is completely different, during the PM programme, couples were not guided to touch the intimate areas of the body. However, here a question emerges: would the PM programme, a non-sexual education programme, and non-sexual massage such as PM be able to support couples who experienced sexual dissatisfaction by raising relationship satisfaction? The question is worth exploring in the future.

Couples experienced PM as holistic stress relief, and such a qualitative finding supports the quantitative data reported previously [28,38]. Similarly, these reports are aligned with the positive physiological changes that have been reported as a consequence of decreased cortisol [37], adrenocorticotropic hormone [56] and norepinephrine levels, and increased dopamine and serotonin levels [57], as well as the “relaxing/love hormone” oxytocin [56]. Recent neuroscience also has found that C tactile afferents mediate oxytocin release during pleasant tactile interactions [58,59]. In the system of stress reaction, cognitive stimuli (physical and emotional) affect the brain, hormone balance, and immune cells [60]. Current concepts of stress take account of neuroendocrine changes as well as hormones and neural activities. The mechanism of massage may be explained through pressure receptors in the skin that stimulate cerebral blood flow and activate the parasympathetic nervous system, which brings a feeling of relaxation and wellbeing [61,62]. These theories may explain the feelings of stress relief physically, mentally, and emotionally through the experience of PM.

PM was also perceived as a relationship-promotor by giving and receiving tactile pleasure. Couples experienced deeper connection and closeness possibly through sharing the positive feelings of gratitude, love and care through practicing PM. These positive feelings seem to be elicited from the *pleasurable touch* of massage. Previous touch research has found that tactile pleasure may be related to activation of a brain region, the pregenual anterior cingulate cortex [63,64], which activates during pleasant sensations and pleasure [65]. Combined with the release of oxytocin, the neuropeptide related to “social bonding” and “trust” [56,66], these mechanisms might explain the effect of massage on promoting positive relationships and cultivating positive emotions which satisfy the human need to connect [11].

Further, the statement of one of the participants, “you appreciate your partner energy towards the massage and want to give it back.” (P34 D18 F 20′s) is fascinating.

The sense of gratitude and appreciation towards the partner as a massage giver, which was expressed commonly by others in the current study has also been reported in other literature e.g., [67,68]. However, the expression of the wish to *“give it back”* may indicate the *reciprocal effect in kindness* being promoted during massage. It implies that the receiver’s natural feeling of gratitude for *“kindness”* or *“love and care”* that was conveyed from the giver through massage is now stimulating the receiver to be kind in return. In other words, PM seems to create the *chain of gratitude*, which is in line with the theory of wellbeing linking with gratitude [69,70].

Couples also perceived PM as a skill to manage stress, to improve communication as well as a practical reciprocal mutual support, namely a *selves-care* skill. The PM programme seems to provide not only massage skills but also both verbal and non-verbal communication skills. Massage itself generally represents a non-verbal communication in the massage literature e.g., [71,72]. Massage has the capacity and a role to interact between giver and receiver e.g., delivering care and love [72], and also intrapersonal e.g., self-awareness, interoceptive [73,74]. In the current study, couples enjoyed both the non-verbal pleasant touch that delivers care and love without words, and verbal communication in the learning process of PM. To understand the partner’s need, couples were encouraged to communicate verbally, especially in the early stages of learning. Interestingly, participants commonly expressed communication regarding not only the improvement itself, but also the awareness of its importance (i.e., the necessity of improvement) during PM, e.g., *“however perhaps I should be better at telling him what I like too.” (P29 D16 F 20′s)*. Effective communication is an important factor in *selves-care* skills. The *selves-care* skill also seems to provide a sense of self-efficacy. Self-efficacy has previously been found to be deeply connected with individual wellbeing [47]. As mastery experiences and reduction of stress reactions contribute to an individual’s self-efficacy [47], acquiring PM skills, being able to apply them and seeing the results may have influenced the couples’ confidence. These findings are consistent with the caregiver’s perception of self-efficacy [67,68].

However, learning new skills generally requires time and dedication. The perception of a time commitment may be related to individuals viewing PM either as a benefit or a burden. It is possible that couples who perceived PM positively may have more personal resources such as available time to dedicate to the learning and practice required [29]. In any event, both learning new skills and building relationships without the investment of time may be impossible in the human world. Couples who are more aware of this and adjust their lives accordingly may be able to better appreciate “quality time together”.

**Figure 3:**
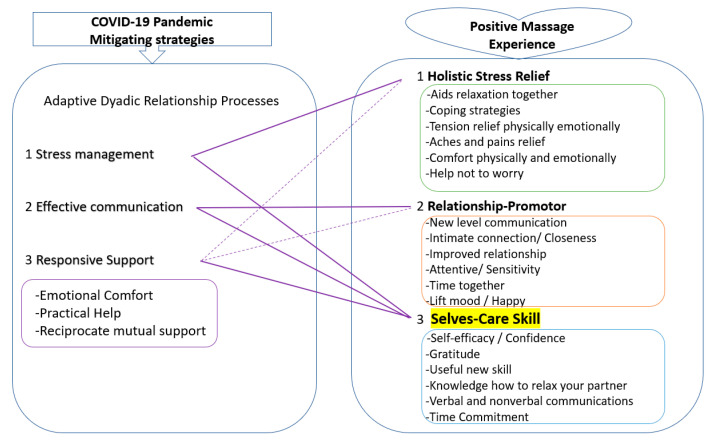
Theoretical link between suggested mitigating strategies by Pietromonaco and Overall [12] and the Positive Massage experience. Purple solid line: Direct link, Purple dotted line: Partial link.

Interestingly, and as presented in Figure 3, the themes identified here are closely linked with the suggested COVID-19 mitigating strategies suggested by Pietromonaco and Overall [12]. Their strategies include: coping with all the external and internal stressors; effective communication; responsive support; and effective support exchanges. The keys of communication are to understand each other’s perspective and respond to each other’s needs, which would require sensitivity and attentiveness in attitude. Responsive support would mitigate any stressful impact by aiding a partner’s feelings of being connected and supported, especially when such support is tailored to the partner’s contextual and personal needs such as emotional comfort, practical help, or building individual capability [12]. Reciprocal mutual support between partners was particularly recommended, because just receiving support may be linked with a negative mood [75]. All of these suggestions have a surprisingly good fit with the findings from the participants’ experiences of PM.

In particular, the *selves-care* skill is distinctive because as shown in Figure 3, it links to all the suggestions of stress management, effective communication, and responsive support. The concept of *selves-care* is new [28] but important. A partner’s wellbeing directly affects that of their correspondent, although western individualism tends to focus on the individual in even the specialist science of wellbeing e.g., [41,42]. One of the core characteristics of *selves-care* skill is reciprocal mutual support, a process that has been shown to buffer negative emotions among couples under stressful life transitions [75]. Furthermore, PM can create *a reciprocal effect in kindness*, as stated earlier. PM possibly promotes a *chain of gratitude*, care, connection, and communication. Thus, PM can be an effective *selves-care* skill to protect couples’ wellbeing through holistic stress relief and by promoting relationship quality.

**Figure 4:**
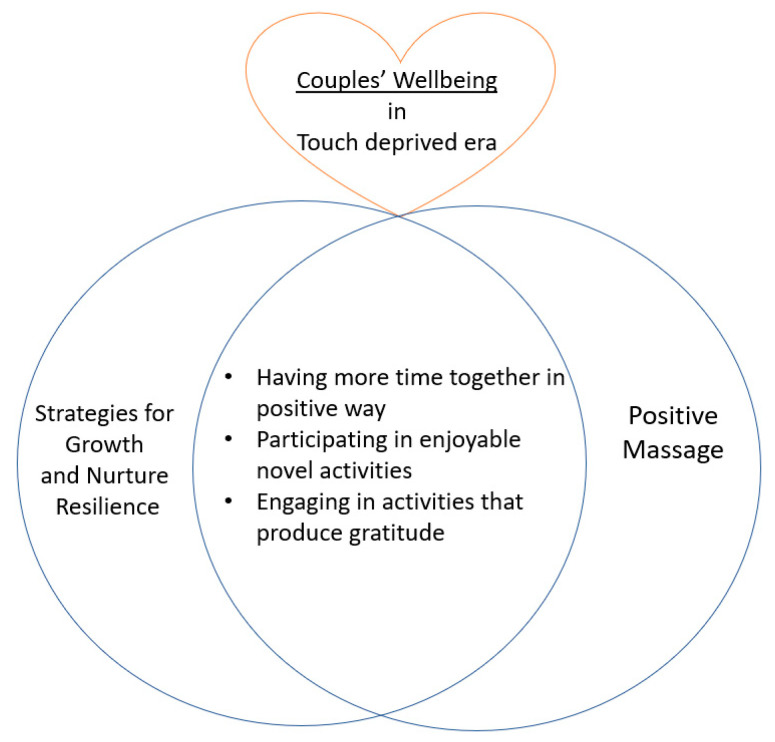
A Perfect Fit: The link between the recommended strategies for the relationship growth during COVID-19 [12] for resilient couples and Positive Massage.

This article is unique in presenting a way to use massage as a preventative intervention for healthy but stressed couples, rather than the therapeutic use of massage for ill populations. For healthy resilient couples, stressful times such as the COVID-19 pandemic can be an opportunity to learn and practice something beneficial, and to incorporate it into their daily life. Interestingly, ‘Danger’ in Japanese consists of two letters: one means ‘danger’, and the other means ‘chance/opportunity’. In this regard, the characteristics of PM fit well with the strategies for resilient couples recommended by Pietromonaco and Overall [12] (Figure 4). Creating quality time together by sharing positive experiences such as taking part in enjoyable novel activities [12] that would create appreciation and gratitude [76] is exactly what PM can provide according to the findings of the current study.

Additionally, an interesting pattern has been revealed throughout the couples’ experiences. The three themes identified were expressed in an intertwined way, which is also depicted in Figure 2. This might demonstrate that the characteristics of massage are multidimensional and inextricably related to benefits for wellbeing, e.g.,

*“It was a wonderful experience that has taught us skills to help each other feel good and relax and has actually brought us closer together which I didn’t think was possible!“ (P16 D9 F 20′s)*

### 4.1. Implications and Applications

The current qualitative data from partners’ experiencing reciprocal massage indicates five possible implications. Firstly, the concept of *selves-care* is new but important, therefore it should be promoted for couples’ relational and individual wellbeing. Secondly, the reciprocal massage can be a practical manifestation of *selves-care*. By using massage as an intervention, the current article has shed new light in the field of couples’ relationship and wellbeing. Most interventions in the field have been verbal communication strategies or programmes using cognitive and emotional therapeutic approaches. Conversely, the use of a pleasant tactile intervention such as PM is a novel approach. Using pleasurable massage aiming for prevention and mitigation of negative outcomes is both new and innovative. Thirdly, reciprocal massage can be promoted in the real world as a home-based *selves-care* skill following short training such as the PM programme. Using reciprocal massage as an intervention is again a novel and new approach in massage research science. The focus of most massage research to date has been on the benefit of receiving massage via a specialist practitioner. The current authors hope it will expand the massage researchers’ view regarding the possible efficacy of reciprocal massage such as PM for the benefit of couples’ personal and relational wellbeing. Fourth, the current article extends lay massage science, for which there is currently a paucity of exploration. Especially in Western academia, the study of lay massage in the literature is still rare. Lastly, the current study has opened a new door to the reciprocal use of massage in close relationships. Such home-based massage may be promoted to the wider population incorporating the family, support bubbles, and other close relationships for use both during a stressful pandemic and after it. In sum, the positive findings reported here suggest that such a pleasant tactile intervention for couples’ wellbeing might be widely promoted as a *selves-care* modality and incorporated into daily life, particularly in a touch-deprived period such as Covid-19.

### 4.2. Limitations and Further Study Recommendations

The experiences reported here were collected from generally “stressed” couples rather than those living in times of COVID-19. Therefore, it is difficult to extrapolate them to life during a specific stressful pandemic. One important shortcoming was that the qualitative data collected online was in conjunction with quantitative assessments, therefore a lack of depth and detail may be provided in participants’ responses. A qualitative study using semi-structured interviews would elicit a deeper context of the experiences. An RCT evaluation incorporating self-efficacy and resilience would equally add value to the lay massage literature. In fact, an RCT study was planned for 2020; however, it was not able to start due to the restrictions imposed on face to face research by the pandemic. Possible bias also exists since the current first author provided the PM programme and had direct contacts with participants; therefore, these participants may have given favorable responses, although data collection was online at home, distant from the direct presence of the authors. A further shortcoming was that evaluation of the potential long-term effects of PM was not included. Hence, we propose the need for future work to consider both the short- and long-term effects of PM on couples during normal conditions and indeed in a more stressful environment where possible. Quantitative and qualitative studies to explore the effects of the PM programme for couples who experience sexual health implications or dissatisfaction on their wellbeing including relationship satisfaction would be interesting. Investigating the effects of PM in other close relationship (e.g., families and supporting bubbles) may also be beneficial in this regard, potentially identifying which populations would gain the most benefits from PM.

Additionally, because of the difficulties that some participants expressed, contraindications of PM should include when either/both are under time pressure and when either/both are overtired as well as when one has a fever or is unwell. To avoid frustration, couples should be advised not to expect high performance from the partner (e.g., in the massage technique), especially whilst undertaking the PM programme.

Finally, there was a suggestion from some participants to deliver PM just giving or receiving on alternate days rather than exchanging on the same day:

*“It is easier giving and receiving massage is different day and time (not giving and receiving at the same time). When I am tired, I am happy to receive massage but not happy to give, vice verse.” (P14 D8 F 50′s)*

*“after relaxing from just received massage, you get tired again from giving the massage” (P34 D18 F 20′s)*

A future study should consider this suggestion.

## 5. Conclusions

This article focused on couples prior to the onset of COVID-19 and argued that the effects of accommodating a relationship-based intervention using a dyad warm tactile touch modality may have wide ranging potential benefits for touch deprived era such as during and after the pandemic. This qualitative study of the experience of PM revealed that stressed but healthy couples perceived exchanging massage as delivering holistic stress relief, relationship-promotion, and *selves-care* skills to relax both the partner and the self by creating positive feelings such as relaxation, love, care, and gratitude. This is aligned with the lay massage literature that has shown physical, psychological, and relational benefits for both receivers and givers. Learning new skills was seen as challenging in terms of time and skill by a few; however, couples mostly enjoyed the experience and the time to learn and practice mutual massage together. The *selves-care* skill seems to provide couples with coping strategies to relieve tension physically, mentally, and emotionally that elicited self-efficacy, as well as promoting relationships via new levels of communication, deeper connection, and sense of gratitude through spending quality time together.

This article proposes that the positive perceived experiences of PM fit well with the mitigation strategies proposed by Pietromonaco and Overall [12] against the possible major negative impacts of COVID-19 on personal and relational wellbeing. For resilient couples, the restrictions of COVID-19, lockdown and isolation can be turned into an opportunity to share quality time together, and use it to learn pleasant and gratitude-focused skills that incorporate the health-promoting behaviour of exchanging massages in daily life. As PM has a core value of *pleasurable touch* that can meet the most basic human need for touch and connection, it may be appreciated not only by couples but in other close relationships such as families and supporting “bubbles”, particularly at times when individuals are at risk of extensive deprivation of touch.

In summary, couples perceived Positive Massage as an effective *selves-care* skill providing holistic stress relief and relationship-promotion. Learning together and reciprocal massage facilitated the ability to relax one another via verbal and nonverbal communication, promoting gratitude, love, care, deeper connection, and self-efficacy through quality time together and *pleasurable touch*. Promoting the concept of *selves-care* and the practical application of reciprocal lay massage such as Positive Massage as a *selves-care* skill would be beneficial for couples’ personal and relational wellbeing.

## Figures and Tables

**Table 1:** Demographic data.

Number of Participants	Total
Started	42 (group A = 24; B = 18)
Completed	38 (group A = 20; B = 18)
Data recorded	34 (group A = 17; B = 17)
Mean Age	36.8 (SD 10.3)
**Gender**	
Male	15
Female	19
Sexual Orientation	
Heterosexual	28
Homosexual	6
Mean Time length of relationship/Year	8.3 (SD 9.6)
**Marital status (% within group)**	
Married	16 (47.1%)
Cohabitant	8 (23.5%)
Other	10 (29.4%)
**Ethnicity (% within group)**	
White British	21 (61.8%)
White European	6 (17.6%)
Asian	2 (5.9%)
Other	2 (5.9%)
Prefer not to state	3 (8.8%)

## Data Availability

The raw data are available from the first author if requested.
